# A Review of Cinnamic Acid’s Skeleton Modification: Features for Antibacterial-Agent-Guided Derivatives

**DOI:** 10.3390/molecules29163929

**Published:** 2024-08-20

**Authors:** Rose Malina Annuur, Desita Triana, Teni Ernawati, Yuta Murai, Muhammad Aswad, Makoto Hashimoto, Zetryana Puteri Tachrim

**Affiliations:** 1 Research Center for Pharmaceutical Ingredient and Traditional Medicine, National Research and Innovation Agency, Kawasan Sains Teknologi (KST) BJ Habibie, Serpong, South Tangerang 15314, Indonesia; 2 Division of Applied Bioscience, Graduate School of Agriculture, Hokkaido University, Kita 9, Nishi 9, Kita-ku, Sapporo 0608589, Japan; 3 Faculty of Pharmacy, Hasanuddin University, Makassar 90245, Indonesia; aswadfar@unhas.ac.id

**Keywords:** cinnamic acid, antimicrobial, antibacterial, Fischer esterification, Steglich reaction, amidation

## Abstract

Antimicrobial resistance has emerged as a significant danger to global health, and the need for more effective antimicrobial resistance (AMR) control has been highlighted. Cinnamic acid is abundant in plant products and is a potential starting material for further modification, focusing on the development of new antimicrobial compounds. In the following review, we describe the classification of critical antibacterial-guided reactions applied to the main skeleton structure of cinnamic acid derivatives over the last decade. Of all of the main parts of cinnamic acids, the phenyl ring and the carboxylic group significantly affect antibacterial activity. The results presented in the following review can provide valuable insights into considerable features in the organic modification of cinnamic acids related to antibacterial medication development and the food industry.

## 1. Introduction

At present, the most important global health issue is antimicrobial resistance (AMR). Antimicrobial resistance has emerged as a significant danger to global health, and to solve this issue directly, multiple organizations must work in coordination. Antimicrobial drugs can fail to prevent the formation of resistant microbes due to mutations or the acquisition of mobile genetic elements containing resistance genes. The requirement for more effective AMR control has been highlighted by the World Health Organization, which recognizes AMR as a global threat to the discovery of novel antibiotic targets [1,2]. A significant treatment issue has emerged from the extensive usage of several antibiotic classes and the development of methicillin-resistant *Staphylococcus aureus* (MRSA). A concerning issue impacting humans is antibiotic resistance [3]. Antibiotic resistance is a complex and relatively new phenomenon with many causes. The first contributing aspect is the sharp decline in the study and creation of novel antibiotics. The emergence and dissemination of drug-resistant or even multidrug-resistant microorganisms is the second contributing factor [4]. Antibiotic resistance can also be promoted by improper use of antibiotics without a valid prescription in some developed countries. Moreover, antibiotic pollution, the term used for excessive usage of antibiotics, leads to higher antibiotic concentrations in the environment, including the addition of antibiotics to agricultural feed. Gene transfer and microbial behavior are expanding globally as a result of these processes [5].

**Figure 1:**
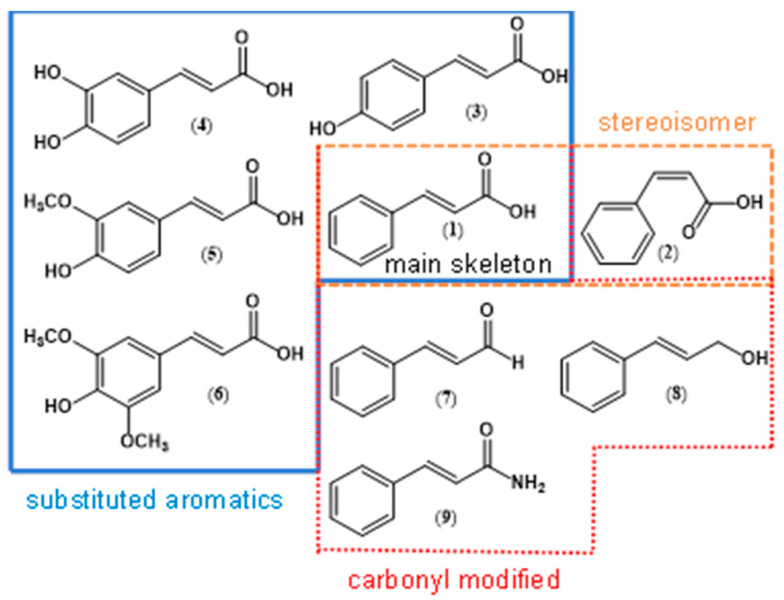
Derivatives of cinnamic acid from nature: *trans*-cinnamic acid (**1**), *cis*-cinnamic acid (**2**), *p*-coumaric acid (**3**), caffeic acid (**4**), ferulic acid (**5**), and sinapic acid (**6**), cinnamaldehyde (**7**), cinnamyl alcohol (**8**), and cinnamamide (**9**).

Cinnamic acid, or carboxylic acid, is abundant in plant products such as whole grains, fruits, vegetables, honey, and beet [6]. Cinnamic acid has a *trans* (**1**) or *cis* (**2**) (Figure 1) configuration based on whether an acrylic acid group substitutes the phenyl ring, with the latter being the more ubiquitous of the two. Although the *cis* form is recognized, it is typically found in nature in the *trans* configuration. Phenylalanine ammonia-lyase promotes *trans*-configuration production from phenylalanine [7]. The most common cinnamic acid derivatives found in nature are *p*-coumaric acid (**3**) [8], caffeic acid (**4**) [9], ferulic acid (**5**) [10], and sinapic acid (**6**, Figure 1) [11]. The amount of cinnamates contained in food and drink has been summarized in other studies [12]. Cinnamic acid derivatives have been shown in several reports as antioxidant [13,14,15,16], anticancer [17,18,19,20,21], antidiabetic [22,23,24], anti-inflammatory [25,26,27,28,29], antifungal [30,31,32,33], antiosteoporosis [34,35], or antimalaria [36,37,38] properties. The cinnamic acid compound has attracted significant attention from researchers over the last three decades. Based on data held in the Scopus database, there were 1368 publications between 1990 and 2024 with the terms “antimicrobial” and “cinnamic acid” included in their title. When a search limit was applied, 249 review titles were found (Figure S1, Supporting Information). Researchers are presently exploring cinnamic acids in a wide range of fields [39,40,41].

In 2011, De et al. conducted a review of cinnamic acid derivatives as anticancer agents from both natural resources and synthetic processes [42]. Sova, in 2012, reported the antioxidant and antimicrobial activities of natural and synthetic cinnamic acid derivatives [43]. In the same year, another group also reported the application of cinnamic acid derivatives in the treatment of tuberculosis, malaria, and cardiovascular disease. The authors provided a literature review on the synthesis of different cinnamic acid derivatives from amides, hydrazides, esters, and other related derivatives [44].

Moreover, in 2014, Guzman presented a review of natural cinnamic acids, synthetic derivatives, and hybrids with antimicrobial activity. The authors of this particular review focused on chemical substances demonstrating fungal or bacterial growth inhibition and carrying the cinnamic skeleton. Studies on the mechanism of action, structure–activity relationships, and whole-cell inhibitory efficacy are highlighted [45]. Cinnamamides (amide of cinnamic acid) were summarized in terms of their biological activity by Gaikward and co-workers. The authors reviewed the binding interaction and structure–activity relationships of cinnamamide derivatives [46]. Our group also previously explored using cinnamic acid derivatives as α-glucosidase inhibitor agents from natural and synthetic compounds [47]. In their recent study, Deng et al. reviewed the application of cinnamic acid in the structural modification of natural products. The authors focused on the natural products of cinnamic acid derivatives and the theoretical basis of their biological activities [48]. In 2023, Kernou et al. focused on the review of rosmarinic acid ester-type cinnamic acid derivatives between 3,4-dihydroxyphenyllactic acid and caffeic acid. Rosmarinic acid was first found in nature by Scarpati and Oriente in 1958 from *Rosmarinus officinalis*. The authors of this review discuss its applications for the treatment of microbial pathogens [49].

In their study, Chen et al. summarize the reactions used in the synthesis of cinnamic acid derivatives over the last decade (2010–2020) [50]. A review of the reaction and modification of cinnamic acid derivatives leading to its bioactivity, mainly as an antibacterial, has yet to be performed. In the following review, the important antibacterial-guided reactions that apply to the main skeleton structure of cinnamic acid derivatives are extensively classified. The features of this typical modification in cinnamic acid derivatives are emphasized for its application as an antibacterial agent. Through this review, we aim to cluster the reactions that occur in cinnamic acid’s main skeleton (**1**) (Figure 1), at least over the past decade, through its function as an antibacterial-agent-guided derivative. Other cinnamic acid derivatives (**2**–**9**) from stereoisomer, substituted aromatics, and carbonyl modification will be described in the following section.

Commonly used antibacterial techniques include broth or agar dilution and disk-diffusion methods. Through this widely used technique, a standardized inoculum of the test microorganism is used to inoculate agar plates. Subsequently, the agar surface is covered with filter paper disks, each measuring roughly 6 mm in diameter and holding a desired concentration of the test compound. An antimicrobial drug diffuses into the agar, preventing the test microorganism from germinating and growing. The widths of these inhibitory growth zones are then determined. Furthermore, due to the difficulty of measuring the amount of the antimicrobial agent that diffuses into the agar medium, the agar disk-diffusion method is unsuitable for determining the minimum inhibitory concentration (MIC). Since dilution procedures enable one to calculate the concentration of the tested antimicrobial agent in the agar (agar dilution) or broth medium (macro-dilution or micro-dilution), these methods are the most suitable for determining MIC values [51].

## 2. Natural Resources of the Antibacterial Activity-Guided Derivatives of Cinnamic Acid

The development of cinnamic acid derivatives to resolve the issue of antimicrobial resistance can be carried out following the determination of the antimicrobial activity of its derivatives from natural resources. In advance, as cinnamic acid is abundant in nature and acts as a potential starting material for further modification, the use of cinnamic acid derivatives as antibacterial agents from natural resources is summarized in the present section. The spice cinnamon (*Cinnamomum zeilanicum*), which is used as a food additive with known antibacterial and insect-repellent effects, is the source of the word “cinnamic”. Other common natural sources of cinnamic acids include coffee beans, tea, cocoa, pears, apples, citrus, berries, grapes, brassica vegetables, spinach, beetroot, tomatoes, potatoes, celery, and cereals [45].

Propolis, which also includes secondary metabolites that may affect antibacterial activity (flavonoids), possesses significant antibacterial characteristics, mainly due to the presence of cinnamic acid derivatives [52,53]. Employing the disk-diffusion method, *Trans*-cinnamic acid (**1**) was evaluated to determine its antimicrobial activity. The measuring scale was described as follows (including the disk diameter): <20 mm indicates a highly inhibitory zone; <20–12 mm indicates a moderately inhibitory zone; and <12 mm indicates no inhibitory zone. Furthermore, overly aquacultured fish are challenged by a variety of stressful conditions, including low water quality and high stock density. Fish diseases can spread quickly, depending on the environmental factors involved. Compound (**1**) inhibited *Aeromonas sobria* and *Aeromonas salmonicida*, *Vibrio crassostreae*, *Vibrio* (*Listonella*) *anguillarum*, and *Yersinia ruckeri* bacteria that cause fish diseases at the zone of inhibition range of 12–14 mm [54]. Furthermore, the geometric isomer of the structure, *cis*-cinnamic acid (**2**), demonstrated a minimum bactericidal concentration (MBC) of 2.5 μg/mL, which was more potent than *trans*-cinnamic acid (**1**) (300 μg/mL) against *Mycobacterium tuberculosis* [55].

Additionally, *p*-Coumaric acid (**3**), a *p*-hydroxyl group substituted aromatic of cinnamic acid, has been demonstrated to be a key antimicrobial agent against *Escherichia coli*, *Bacillus subtilis*, *Lactobacillus plantarum*, and *Lactobacillus hammesii*. In a study, reducing the number of hydroxyl groups or substituting hydroxyl groups with methoxy groups was found to have no significant effect on antibacterial activities [56]. In comparison, *p*-coumaric acid (**3**), caffeic acid (**4**), and ferulic acid (**5**) presented the highest inhibition at a concentration of 2.0 mM against *Pseudomonas syringe*, *E. coli*, and *B. subtilis*. These results indicated that the antibacterial characteristics fit into the skeleton of *p*-coumaric acid and required no additional hydroxyl groups in the aromatic moiety of cinnamic acid [57]. The authors of a previous report also noted that ferulic acid (**5**) exhibited antimicrobial activity against *Cronobacter sakazakii* strains. Based on the utilization of the agar dilution technique, the minimum inhibitory values were found to be between 2500 and 5000 μg/mL [58]. Caffeic acid (**4**) against pathogen bacteria *M. tuberculosis* and *Klebsiella pneumoniae* showed inhibition with a minimum inhibition concentration (MIC) of 64–512 μg/mL. The authors also tested pomegranate extracts, which contained a mixture of phenolic compounds such as caffeic acid (**4**), ellagic acid, epigallocatechin-3-gallate, and quercetin. Pomegranate fruit pericarp extracts in methanol and water exhibited higher antitubercular activity with MIC values of 64–512 and 64–1024 μg/mL, respectively [59]. In general, the antibacterial activity of substituted aromatic in cinnamic acid is in the order of *p*-coumaric (**3**) > caffeic (**4**) > ferulic acid (**5**).

Malheiro et al. investigated the effects of cinnamic acid (**1**) and cinnamaldehyde (**7**) on the growth of *E. coli*, *Staphylococcus aureus*, and *Enterococcus hirae* (Figure 1). Their results showed that at concentrations of 3–8 mM, cinnamaldehyde (**7**) had greater potential to inhibit the growth of all bacteria and at concentrations of 8–10 mM for *S. aureus* than cinnamic acid (**1**) (MBC >25 mM). Cinnamyl alcohol (**8**) decreased the growth of *E. coli* (8–15 mM) and *S. aureus* (20 mM); in comparison, cinnamamide (**9**) at concentrations of 20–25 mM affected the growth of *E. coli*. Additionally, their structure determined the antibacterial activity of cinnamic acid derivatives. Cinnamaldehyde (**7**) was found to possess more potential antibacterial agents than compounds (**1**,**8**,**9**) [60]. The authors of a number of leading reported studies also described the benefits of cinnamaldehyde [61] and cinnamic acid derivatives [62], with a previous focus on in vitro and in vivo antimicrobial activity [63].

## 3. Classification of the Features of the Organic Modification of Cinnamic Acid Derivatives as an Antibacterial Agent

A number of researchers are interested in developing cinnamic acid derivatives because their main skeleton has three active-site groups: phenyl, double bond, and carboxylic groups. The development of cinnamoyl-based amides with different substituents at the phenyl ring against colorectal cancer (CRC) cells has been encouraged by the discovery of potential anticancer medicines [64]. In their study, de Morais et al. also prepared cinnamic acid and its corresponding acid chloride, cinnamoyl chloride, to examine antileishmanial activity [65]. Since cinnamic acid can be transformed through several processes, the classification of the present modification of its main skeleton can, therefore, be described herein.

### 3.1. Fischer Esterification

**Figure 2:**
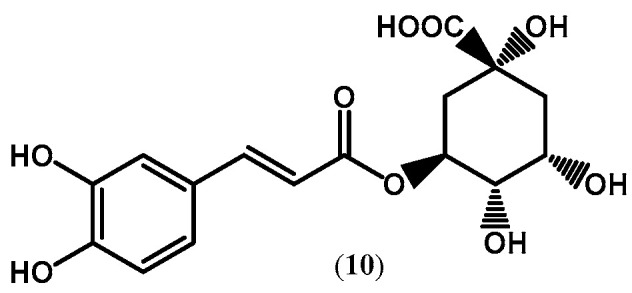
Structure of chlorogenic acid (3-*o*-caffeoylquinic acid) (**10**).

Chlorogenic acid (3-*o*-caffeoylquinic acid, **10**, Figure 2) is a natural ester of hydroxycinnamic acid that is commonly found in coffee or black tea. The antibacterial activity of pure chlorogenic acid (**10**) was investigated by Li et al. in their study. Chlorogenic acid (**10**) possesses a MIC range of 2500–20,000 μg/mL against eight strains of *S. aureus* [66]. Our group successfully isolated methyl cinnamate (**11a**) from *Alpinia malaccensis* oil through steam distillation. Thereafter, compound (**11a**) was evaluated at MIC ranges of 2000–4000 μg/mL against methicillin-resistant *S. aureus*, *B. subtilis*, *P. aeruginosa*, *E. coli*, and *S. aureus* [67]. Many researchers have attempted to identify potential ester compounds from the skeleton of cinnamic acid and ester groups as antibacterial agents. The process of esterification involves reacting alcohol with carboxylic acid to generate an ester molecule, replacing the OH group on the carboxylate group with an alkyl alcohol group. Alternatively, carboxylic acid and an alcohol react with an acid in a process known as Fischer esterification. An acid catalyst facilitates the alcohol’s nucleophilic attack on the carboxylic acid’s carbonyl carbon [68], and this typical esterification is then clustered as the synthesis of cinnamic acid derivatives under the Fischer esterification process.

The results of the synthesis of ester cinnamic acid are presented in Table 1. Compounds (**11a**–**c**) with hydrogen substituents were found to have low activity against all bacterial strains, including *S. aureus*, *S. epidermidis*, and *P. aeruginosa*, at a MIC of 128–512 μg/mL (Table 1, entry 1) [69]. When the hydroxyl (**12a**–**c**) was attached to the phenyl ring, antibacterial activity was shown at a MIC range of 0.98–2.68 μg/mL. However, the chloro (**13a**–**c**) substituent showed no activity (Table 1, entries 2, 3) [70,71]. In comparison, for two other substituents that attach to the phenyl ring (**11d** and **13d**), caffeic acid derivatives (**12d**) with the butyl group showed good activity against *S. aureus* and *E. coli* (MIC 0.89 μg/mL) (Table 1, entries 4–6) [69,70,71]. Compound (**11e**) exhibited low activity against *S. epidermidis* and *P. aeruginosa* at concentrations of 128 μg/mL compared with amoxicillin (no microorganism growth, Table 1, entry 7) [69]. Moreover, compound (**12e**) demonstrated significant potential to inhibit *S. aureus* and *E. coli* at 1.60 and 0.80 μg/mL, respectively (Table 1, entry 8) [70]. Compound (**11f**) inhibited *S. aureus*, *S. epidermidis*, and *P. aeruginosa* at 128 μg/mL (Table 1, entry 9) [69]. Caffeic acid (**12f**) with an isopropyl group provided a MIC value of 2.02 μg/mL against *S. aureus* and *E. coli*. However, compound (**13e**) with a chloro substituent showed no activity against all bacterial strains (Table 1, entries 10, 11) [70,71]. Compound (**11g**) did not affect all strains. However, compound (**12g**) at a MIC range of 1.60–3.17 μg/mL inhibited *S. aureus* and *E. coli* (Table 1, entries 12,13) [69,70]. The methoxyethyl group (**12h**) showed higher activity than (**13f**), with a MIC range of 3.52 μg/mL against *S. aureus* and *E. coli* (Table 1, entry 14, 15) [70,71]. Based on the above results, caffeic acid derivatives (**12a**–**h**) indicated greater potential as antibacterial agents against the *S. aureus* and *E. coli* strains. The hydroxy group on the phenyl enhanced the antibacterial activity of the cinnamic acid derivatives. The presence of bulky and lipophilic short-chain groups likely reduced the antibacterial activity of the corresponding compounds.

### 3.2. Alkyl Halide Reagent

An ester group is also produced when an alkyl halide and a carboxylate nucleophile react. This reaction includes the substitution of an alkyl halide with an oxygen nucleophile via backbone attack at the acyl carbon. This S_N_^2^ process, in normal circumstances, requires a suitable leaving group on an unhindered carbon (tosylate or halide). The resonance-stabilized carboxylate is not highly nucleophilic; hence, steric hindrance may cause this reaction to be particularly sluggish [68]. For Fischer esterification, most carboxylic acids are acceptable; however, primary or secondary alcohol is usually preferred. However, esterification via alkyl halide can facilitate the formation of various esters. Nevertheless, increasing the molecule’s lipophilicity led to decreased activity, and in compounds (**14a**,**b**), no inhibitory action appeared against the *P. aeruginosa* and *S. aureus* strains (Table 2, entries 1, 2) [71]. The compound decyl cinnamate (**15a**) exhibited activity at concentrations of 128 μg/mL against *S. aureus*, *S. epidermidis*, and *P. aeruginosa* (Table 2, entry 3) [69]. Moreover, compounds (**14c**,**d**) and (**15b**) with a benzyl group bearing chloro and methoxy substituents had no significant impact on antibacterial properties at the para position (Table 2, entries 4–6) [69,71].

### 3.3. Modification via Acid Chloride

**Scheme 1:**
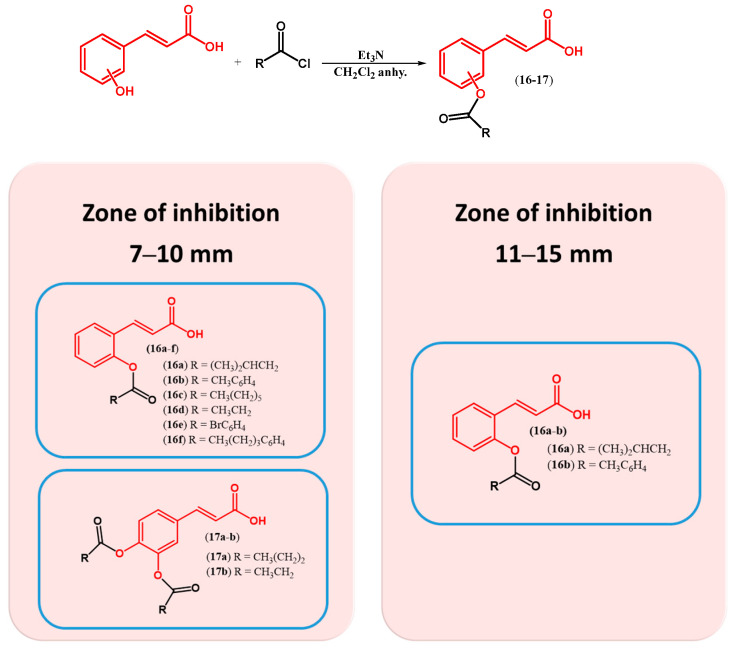
Esterification of coumaric and caffeic acid derivatives (**16**–**17**).

Modification of the cinnamic acid skeleton via acid chloride to produce cinnamate esters can involve two possible pathways: (a) the reaction of the hydroxyl(s)-substituted aromatic moiety of cinnamic acid derivatives (coumaric (**3**) or caffeic acid (**4**)) with contributed acetyl chloride; (b) the reaction of cinnamoyl chloride derivatives with alcohols or amines. When cinnamic acid reacts with acetyl chloride, the hydroxyl group in the aromatic ring of the cinnamic acid undergoes deprotonation, subsequently leading to a nucleophilic attack on the carbonyl carbon of the acetyl chloride. Typically, the R substituent group of acetyl chloride is opposed by the longer carbon chain or steric attack from the hydroxyl substituent(s) in the aromatic moiety of cinnamic acids. Under basic conditions, the hydroxyl group in the aromatic ring is more nucleophilic than the hydroxyl group in the carboxylic group due to the electron density of the hydroxyl of the aromatic group concentrated on one oxygen. Modification of the hydroxyl substituent in the aromatic moiety of *o*-coumaric and caffeic acid is demonstrated in Scheme 1. The corresponding compounds were tested for antibacterial activity against *Bacillus cereus*, *S. epidermidis*, *S. aureus*, *E. coli,* and *P*. *aeruginosa* using the disk-diffusion technique. Based on the results, all of the products (**16a**–**f**) and (**17a**,**b**) exhibited activity against Gram-positive bacteria, *Bacillus cereus*, *S. epidermidis*, and *S. aureus* (zone of inhibition 7–10 mm). However, only compounds (**16a**,**b**) showed an inhibitory effect against *E. coli* and *P*. *aeruginosa* (zone of inhibition 11–15 mm). Compounds (**16c**–**f**) and (**17a**,**b**) showed no antibacterial activity against all Gram-negative bacteria due to the steric group’s difficulty permeating the double membranes of Gram-negative bacteria [72].

**Scheme 2:**
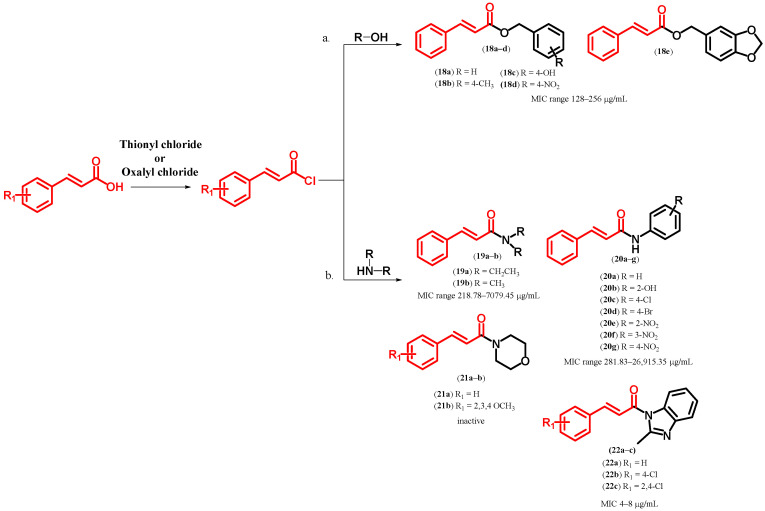
(**a**) Esterification and (**b**) amidation of cinnamic acid derivatives from acid chloride.

A substitution reaction is the method most frequently used to generate ester compounds. An acyl substitution creates an ester from a carboxylic acid, where the –OH leaving group will be replaced with a –OR′ group. Initiating this substitution process involves generating the acid chloride and treating the required alcohol nucleophile in the presence of a base [68]. For example, as shown in Scheme 2a, the esterification of cinnamic acid required thionyl chloride (SOCl_2_) to generate cinnamoyl chloride, which reacted further with the corresponding alcohol to produce the ester. Benzyl cinnamate (**18a**) showed antimicrobial potential at concentrations of 128 μg/mL *S. aureus* and *S. epidermidis*; however, *P. aeruginosa* was less active (MIC 256 μg/mL). The benzyl group bearing electron-withdrawing or -donating groups (**18b**–**d**) had no significant impact on the antimicrobial properties at the para position. Compounds bearing the piperonyl group (**18e**) showed no effect on antibacterial activity [69].

Cinnamic acid (**1**) was converted to cinnamoyl chloride to obtain products (**19a,b**) and (**20a**–**g**) (Scheme 2b). Compound (**19a**), with a MIC of 218.78–7079.45 μg/mL, inhibited all bacterial species; in comparison, compound (**19b**) was less active toward all of the bacteria. Amide compounds from the aromatic amine (**20a–g**) were more active than those from the aliphatic amine (**19a**,**b**). Compounds (**20a**) and (**20b**) with a MIC of 281.83–4786.30 μg/mL showed low antibacterial activity. Compounds (**20c**) and (**20e**–**g**) exhibited potential antimicrobial activity against *E. coli* (MIC of 10,715.19–26,915.35 μg/mL). According to antimicrobial investigations, substituents on the amide nitrogen of the derivatives of cinnamamide contribute to their antimicrobial properties [73]. Fregnan et al. published a report on the synthesis, characterization, and evaluation of piplartine derivatives. However, compounds (**21a**,**b**) showed no activity against *S. aureus*, *E. coli*, and *P. aeruginosa* [74]. Li and co-workers developed novel cinnamic derivatives containing the benzimidazole group and determined their efficacy against *S. aureus* strains. After performing in vitro tests, compounds (**22a**–**c**) with a MIC range of 4–8 μg/mL were found to be more active than the drug ciprofloxacin (MIC of 8 μg/mL) [75].

### 3.4. Steglich Reaction

**Scheme 3:**
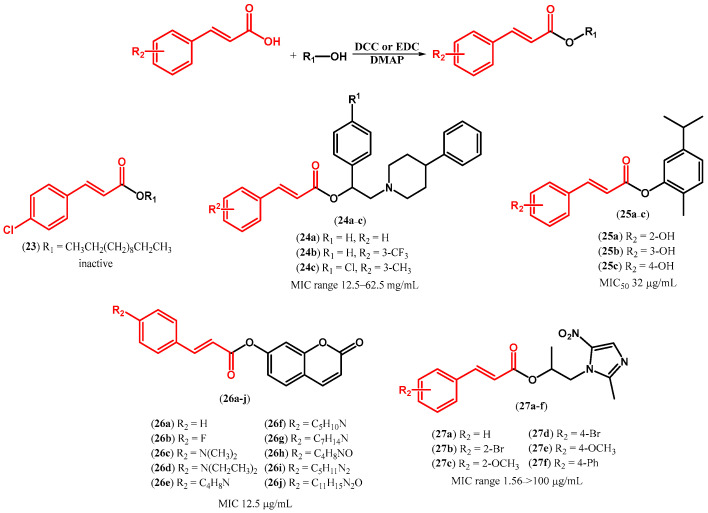
Esterification of cinnamic acid derivatives via the Steglich reaction.

Neises and Steglich demonstrated how catalytic DMAP can significantly increase reactivity in carbodiimide-activated esterification processes. This technique increased product yields, shortened the reaction durations, and made it possible to create compounds that were not accessible at the time [76]. The reaction of cinnamic acid derivatives with the Steglich reagent can be clustered and followed up by several esters of cinnamic acid. The 4-Chlorocinnamic acid derivative (**23**) was synthesized using Steglich esterification (Scheme 3), and its antibacterial activity against *P. aeruginosa* and *S. aureus* strains was tested. However, compound (**23**) with long-chain alkyl showed no inhibition against all bacterial strains [71].

Novel compounds of piperidine-containing cinnamic acid derivatives (**24a**–**c**) were studied for their antibacterial activity against *S. aureus*, *B. subtilis*, *P. aeruginosa*, and *E. coli*. Compound (**24a**) exhibited better activity against Gram-positive bacteria *S. aureus* and *B. subtilis*. However, compound (**24b**) showed potential activity against *P. aeruginosa* and *E. coli* at a MIC range of 25–50 μg/mL. Compound (**24c**) also demonstrated strong efficacy against *E. coli* at 12.5 μg/mL compared to norfloxacin (10 μg/mL, Scheme 3) [77]. *Trans*-cinnamic acid hydroxy-substituted derivatives (**25a**–**c**) were reacted with carvacrol under the Steglich reaction and then identified as potential antibacterial agents (Scheme 3). Compared to compound (**25a**), in which the OH- was in the ortho position, the addition of the -OH moiety in the *meta*- and *para*-positions of aromatic moieties (**25b**) and (**25c**), respectively, resulted in a significant reduction in activity. Product (**25a**, MIC_50_ 32 μg/mL) was the most potent molecule compared with (**25b**,**c**, MIC_50_ > 512 μg/mL) against *Enterococcus faecium*, suggesting that the hydroxyl group at the ortho position is essential for interaction with the bacterial cell wall [78].

Researchers successfully prepared a hybrid compound of coumarin–cinnamic acid derivatives (**26a**–**j**, Scheme 3). They tested its antibacterial properties against *B. subtilis*, *Streptococcus pneumoniae*, *Clostridium tetani*, *E. coli*, *Salmonella typhi*, *Vibrio cholerae*, and *M. tuberculosis* H_37_Rv. Compared with the hydrogen substituent, compounds (**26i**) and (**26j**) demonstrated good activity at minimum bactericidal concentration values ranging from 12.5 to 50 μg/mL against *E. coli*. The interaction of two compounds (**26i**,**j**) with active-site residues of DNA GyrB of *E. coli* resulted in a binding affinity of −8.5 kcal/mol and −8.4 kcal/mol. In comparison, compounds (**26g**) and (**26i**,**j**) with piperidine groups substituted with cinnamic acid showed activity against *M. tuberculosis* H_37_Rv (MBC 25–62.5 μg/mL) [79]. The antibacterial activity of the novel compounds (**27a**–**f**) cinnamic acid–secnidazole was evaluated against *B. subtilis*, *S. aureus*, *E. coli,* and *P. aeruginosa* (Scheme 3). The antibacterial activity of compounds **27a** and **27c** (MIC range > 100 μg/mL) was lower than compound (**27b**) with bromo at the *ortho* position against all bacteria strains (MIC range 25–50 μg/mL). Bromo at the para position (**27d**) enhanced the antibacterial activity at concentrations of 3.13–6.25 μg/mL. Compound (**27f**) demonstrated the most potent biological activity compared to the positive control, Kanamycin (MIC range 1.56–3.13 μg/mL). Substituents at the para position might have slightly enhanced antibacterial properties [80].

**Scheme 4:**
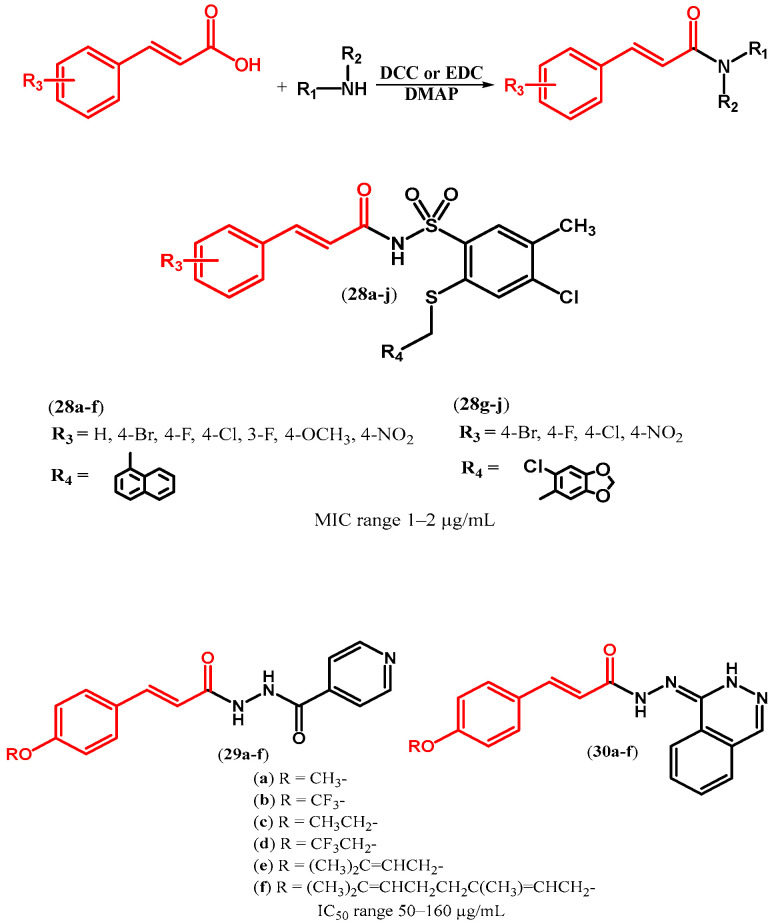
Amidation of cinnamic acid derivatives via the Steglich reaction.

In 2023, Bulakowska et al. successfully developed amide derivatives (**28a**–**j**) *N*-(2-arylmethylthio-4-chloro-5-methylbenzenesulfonyl) with various substituents in the phenyl ring (Scheme 4). They evaluated antimicrobial activity against *S. epidermidis*, *S. aureus*, *E. hirae*, *E. faecalis*, *B. subtilis*, *Corynebacterium diphtheriae*, and *E. coli*. Compounds (**28b**–**d**) showed the most effective activity against *Staphylococcus* sp. and *Enterococcus* sp. in the MIC range of 1–2 µg/mL. Interestingly, the introduction of the different substituents in the aromatic moiety of cinnamic acid and 1-naphthalene in the sulfo bridge of sulfoamide showed higher antimicrobial activity against the tested microorganisms [81]. Substituted cinnamic acid reacted with various hydrazides to gain compounds (**29**,**30**) (Scheme 4). Thereafter, the synthetic compounds (**29**,**30**) were tested to determine their biological activity against *M. tuberculosis* by using a colorimetric microassay. Based on the results, compounds (**29a**) and (**29e**) showed good activity at a half maximal inhibitory concentration (IC_50_) of 50–90 µg/mL. The IC_50_ values for the 1-phthalazine (**30a**–**f**) group were lower than those of compounds (**29a**–**f**); the best compound (**30e**) inhibited *M. tuberculosis* with an IC_50_ of 160 µg/mL [82].

### 3.5. Mitsunobu Reaction

**Scheme 5:**
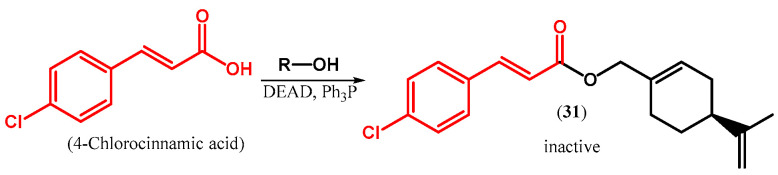
Mitsunobu reaction of cinnamic acid derivatives.

Another commonly used technique is the Mitsunobu reaction; under neutral conditions and at or below room temperature, the reaction between alcohols and carboxylic acids occurs without issue. In most cases, Ph_3_P and DEAD are used to treat a mixture of alcohol and carboxylic acid. The Mitsunobu method has several benefits; however, it has a significant drawback in that it requires a considerable amount of chemicals to be performed, which may make the separation of by-products challenging at times [83]. 4-Chlorocinnamic acid derivatives (**31**) were prepared using the Mitsunobu reaction, which was used to evaluate their antibacterial activity against the *P. aeruginosa* and *S. aureus* strains (Scheme 5). Compound (**31**) had no inhibitory effect [71].

### 3.6. Amidation Using Nitrile

**Scheme 6:**
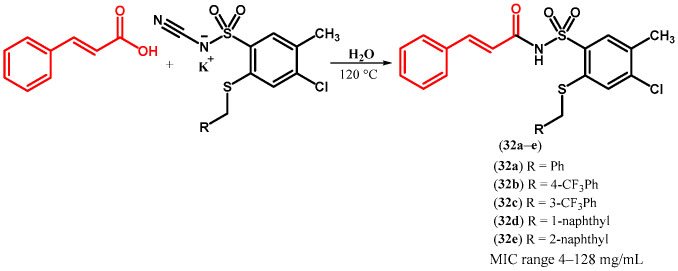
Amidation of cinnamic acid derivatives.

Another method used for the creation of amides devoid of *N*-alkyl groups (RCONH_2_) is to partially hydrate a nitrile (RC≡N). An enol-like intermediate tautomerizes to produce carbonyl when one equivalent of water is added across the nitrile triple bond. This process is comparable to the hydration of an alkyne to produce a ketone product. The nitrile partial hydrolysis reaction conditions are kept relatively mild when the desired amide product is obtained since amides can also hydrolyze to produce carboxylic acids [66]. In 2020, Zolnowska and co-workers synthesized amide derivatives (**32a**–**e**) *N*-(2-arylmethylthio-4-chloro-5-methylbenzenesulfonyl) and examined the antimicrobial activity of each compound on all types of bacteria (Scheme 6). This reaction involved two steps of preparation of starting material *N*-(benzenesulfonyl)cyanamide (**b**). Furthermore, many of these amides prevented *S. epidermidis* and *S. aureus* at MIC values ranging from 4 to 32 µg/mL. However, compounds (**32a**) and (**32e**) showed low activity compared with other amides against *Enterococcus faecalis* at a MIC of 128 µg/mL. Compounds (**32a**–**e**) presented only slight activity against Gram-negative bacteria *P. aeruginosa* and *E. coli* at concentrations of 64–128 µg/mL compared to Gram-positive bacteria [84].

## 4. Utilization of Features in the Modification of Cinnamic Acid Derivatives for Antibacterial Materials

Cinnamic acid can be employed to modify structures, enhancing the properties of lead compounds or potential medications by utilizing the different types of reactions mentioned above. Researchers have expanded its application to include cinnamic acid derivatives. The conventional method of preserving food quality and providing microbiological safety involves adding chemicals like antioxidants and antimicrobials to the food packaging formulation. The food industry is working to create active packaging that can precisely and carefully distribute natural antioxidants [85]. Some researchers have developed biofilms from chitosan, cellulose, or starch-containing natural antioxidant cinnamic acid [86,87,88,89,90,91,92,93]. Furthermore, researchers have expanded its application to include cinnamic acid derivatives as antimicrobial agents. The food industry has recently shown significant interest in antibacterial packaging as a result of the increase in consumer demand for minimally processed, preservative-free goods. Biofilms produced with natural antibacterial agents, such as cinnamic acid derivatives, have shown effectively greater antibacterial activity in studies. Some researchers have focused on the use of cinnamic acid as an antibacterial agent combined with chitosan, cellulose, or starch [94,95,96,97,98,99,100,101].

Steglich esterification is a synthetic method that can be used to modify complicated compounds to cellulose in mild reaction conditions. In the present study, cellulose and cinnamic acid reacted with DIC and DMAP to generate cellulose esters (**33**) (Table 3, entry 1). The antioxidant activity of CNF films was enhanced by esterification with cinnamic acid using carbodiimide based on a 45–80% capacity to scavenge DPPH. The antioxidant activity of CNF films was significantly improved by using this method compared to the original CNF (10% DPPH scavenged activity). Antioxidants can be used in preliminary tests for antibacterial agents for further application [102]. Two distinct reagents, thionyl chloride and EDC, were used for the synthesis of CNF esters of cinnamic acid (**33**) (Table 3, entry 2). The fibers treated with cinnamic acid exhibited activity against *S. epidermidis*. Fibers treated with cinnamic acid via thionyl chloride (TCc) demonstrating no *S. epidermidis* were detected in the sample. Thereafter, via EDCc, the bacterial load was decreased from 9.7 log CFU/mL to 6.9 log CFU/mL compared to CNF (7.1 log CFU/mL). An unsaturated alkyl chain may factor in the cinnamic acid-modified CNF’s apparent antibacterial properties [103].

Chitosan, a biodegradable biopolymer, and its derivatives are used as antibacterial agents—a novel method for treating and preventing biofilms. Cinnamic acid and chitosan are mixed to produce CS-CA compounds (**34**) (Table 3, entry 3). NHS and EDC are chemical agents that are used to create CA-grafted CS. CS-CA derivatives were investigated in vitro on *S. aureus* and *E. coli*. MIC values of CS against *E. coli* and *S. aureus* are 1024 and 2048 µg/mL, respectively. Comparing CS-CA against *E. coli* and *S. aureus* at a concentration range of 256–1024 µg/mL suggests that the derivatives’ antibacterial activity was enhanced with the attachment of CA. The results suggest that CS-CA derivatives may be a promising novel antibacterial agent for the management of infections linked to biofilms. The thickness of biofilms in CS-CA treatment decreased to roughly 16 μm after 24 h of incubation. CS-CA derivatives contribute to antibacterial agents in the food and pharmaceutical industries [104].

Based on the method used in a recent study, compounds from PET-cinnamic acid were generated via the Steglich reaction to prevent the spread of bacteria (Table 3, entry 4). Broth micro-dilution assays were used to determine the MIC of synthetic compounds (**35a**,**b**). None of the tested compounds showed activity against all strains, *S. aureus*, *E. coli*, and *P. aeruginosa,* at concentrations of over 250 µg/mL. The control of gene expression in response to variations in the number of cells in a population is known as quorum sensing. *Chromobacterium violaceum* and *Serratia marcescens*, under quorum sensing (QS) using the agar diffusion method, were used for the inhibition of violacein or prodigiosin production. As a result, only compound (**35b**) showed inhibitory activity, which was indicated by a blurry white halo around the disk at a concentration of 250 µg/disk. Based on the results of molecular docking studies, compound (**35b**) was found to have an energy-binding site for interaction with AbaI (QS signal transduction) −9.5 kcal/mol. It can be concluded that compound (**35b**), which has a ferulic acid component, may impact the QS pathway in the two studied bacteria. The acquired data show that chromophores with a methoxy group added to one part of their cinnamic acid composition work as QS modulators [105].

**Scheme 7:**
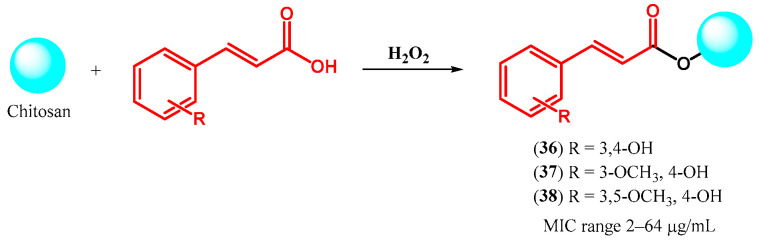
Chitosan reaction with cinnamic acid derivatives.

Chitosan conjugated with cinnamic acid derivatives such as caffeic, ferulic, and sinapic acid were prepared under acidic conditions (Scheme 7). Ascorbic acid in acetic acid solution as a di-acid reacted with hydrogen peroxide (H_2_O_2_) to produce the hydroxyl radical. Subsequently, the hydroxyl and amino groups in chitosan were removed by OH·, leading to the formation of chitosan macro radicals. Chitosan-phenolic acid conjugates are formed when the phenolic acid molecules combine with chitosan radicals. The conjugated derivatives were tested for their antioxidant and antibacterial properties. The unmodified chitosan was found to have an IC_50_ value of 682.95 μg/mL against DPPH; in comparison, that of the conjugates (**36**–**38**) ranged from 135.16 to 381.29 μg/mL. Conjugating cinnamic acid derivatives onto the chitosan enhanced the antibacterial properties of the unmodified chitosan via the dilution method. Examination of ferulic acid-chitosan (**37**) indicated that it shows good potential against *B. subtilis* with a MIC value of 2 μg/mL; however, *B. subtilis* with a MIC value of 64 μg/mL was found in two other conjugates. Conjugate (**37**) showed activity against *Listeria monocytogenes* (MIC of 32 μg/mL) and *E. faecalis* (MIC of 16 μg/mL). The activities of the three conjugates were greater than those of the chitosan (MIC of 128 μg/mL) [106]. Ferulic acid-chitosan (**37**) has been proven to show antibacterial activity against *S. aureus*, in addition to *Morganella morganii* (zone of inhibition range: 31–35.5 mm). However, ferulic acid-chitosan (**37**) had no inhibitory effect against *E. coli* [107].

## 5. Conclusions

**Scheme 8:**
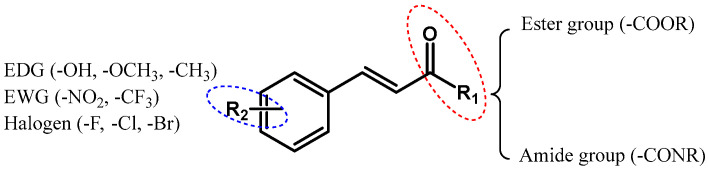
The general structure of modified cinnamic acid derivatives leads to antibacterial agents.

Depending on the substituent R_1_ or R_2_ (Scheme 8), the guidance provided in this review will stimulate the essential features in the modification of cinnamic acid. The substituent R_1_ or R_2_ (Scheme 8) affects the reaction conditions to maintain the cinnamic acid backbone. In typical ester modifications, aliphatic branched or unbranched, aromatic, and nitrogen-containing are the preferred potential antibacterial agents. Furthermore, as shown in the present study, amide modification also showed a similar trend to antibacterial agents. Based on the results of the electron-donating or -withdrawing group-influenced antibacterial activity of cinnamic acid derivatives, there is a limited exploration of substituent R_2_ (Scheme 8) in the phenyl ring. Moreover, the present review can provide valuable insights into considerable features in the organic modification of cinnamic acids related to antibacterial medication development and the food industry.

## Figures and Tables

**Table 1:** Fischer esterification of cinnamic acid derivatives.

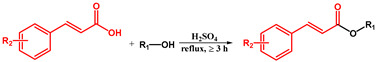						
Entry	Compound	Ref.	R_1_	R_2_	Bacteria	MIC Range (μg/mL)
1	**11a–c**	[69]	CH_3_; CH_2_CH_3_; or (CH_2_)_2_CH_3_	H	*S. aureus*, *S. epidermidis*, and *P. aeruginosa.*	0.98–512
2	**12a–c**	[70]		3,4-OH	*S. aureus* and *E. coli*	
3	**13a–c**	[71]		4-Cl	*S. aureus* and *P. aeruginosa*	
4	**11d**	[69]	(CH_2_)_3_CH_3_	H	*S. aureus*, *S. epidermidis*, and *P. aeruginosa.*	0.89–128
5	**12d**	[70]		3,4-OH	*S. aureus* and *E. coli*	
6	**13d**	[71]		4-Cl	*S. aureus* and *P. aeruginosa*	
7	**11e**	[69]	(CH_2_)_4_CH_3_	H	*S. aureus*, *S. epidermidis*, and *P. aeruginosa.*	0.80–128
8	**12e**	[70]		3,4-OH	*S. aureus* and *E. coli*	
9	**11f**	[69]	CH(CH_3_)_2_	H	*S. aureus*, *S. epidermidis*, and *P. aeruginosa.*	2.02–128
10	**12f**	[70]		3,4-OH	*S. aureus* and *E. coli*	
11	**13e**	[71]		4-Cl	*S. aureus* and *P. aeruginosa*	
12	**11g**	[69]	(CH_2_)_2_CH(CH_3_)_2_	H	*S. aureus*, *S. epidermidis*, and *P. aeruginosa.*	1.60–3.17
13	**12g**	[70]		3,4-OH	*S. aureus* and *E. coli*	
14	**12h**	[70]	(CH_2_)_2_OCH_3_	3,4-OH	*S. aureus* and *E. coli*	3.52
15	**13f**	[71]		4-Cl	*S. aureus* and *P. aeruginosa*	

**Table 2:** Esterification via alkyl halide.

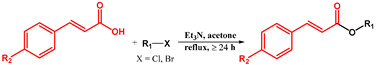						
Entry	Compound	Ref.	R_1_	R_2_	Bacteria	MIC (μg/mL)
1	**14a**	[71]	(CH_2_)_4_CH_3_	Cl	*S. aureus* and *P. aeruginosa*	Inactive
2	**14b**		(CH_2_)_8_CH_3_	Cl		
3	**15a**	[69]	(CH_2_)_9_CH_3_	H	*S. aureus*, *S. epidermidis*, and *P. aeruginosa*	128
4	**14c**	[71]	CH_2_Ph(4-Cl)	Cl	*S. aureus* and *P. aeruginosa*	Inactive
5	**14d**		CH_2_Ph(4-OCH_3_)	Cl		
6	**15b**	[69]	CH_2_Ph(4-Cl)	H	*S. aureus*, *S. epidermidis*, and *P. aeruginosa*	

**Table 3:** Further application of cinnamic acid derivatives.

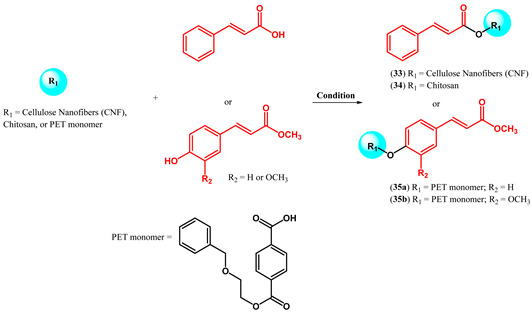				
Entry	Compound	Ref.	Condition	Antibacterial Activity
1	**33**	[99]	DIC, DMAP, at 24 °C for 24 h	
2		[100]	a. SOCl_2_, pyridine, at rt for 24 h	Bacteria load range 6.9–7.1 log CFU/mL of *S. epidermidis* ^a^
			b. EDC, DMAP, at rt for 24 h	
3	**34**	[101]	EDC, at rt for 24 h	MIC range 256–2048 µg/mL against *E. coli* and *S. aureus*
4	**35a**	[102]	DCC, DMAP, at rt for 24 h	Inactive against *S. aureus*, *E. coli*, and *P. aeruginosa*
	**35b**			

^a^ Tested for antioxidants using the DPPH assay.

## Data Availability

Not applicable.

## Supplementary Materials

The following supporting information can be downloaded at: https://www.mdpi.com/article/10.3390/molecules29163929/s1. Figure S1. Scopus data.

## Abbreviations

CA	Cinnamic acid
CNF	Cellulose nanofibril
CS	Chitosan
DCC	*N*,*N*′-Dicyclohexylcarbodiimide
DEAD	Diethyl azodicarboxylate
DIC	*N*,*N*′-Diisopropylcarbodiimide
DMAP	4-Dimethylaminopyridine
DPPH	2,2-Diphenyl-1-picrylhydrazyl
EDC	1-Ethyl-3-(3-dimethylaminopropyl) carbodiimide
Et_3_N	Triethylamine
NHS	*N*-Hydroxysuccinimide
PET	Polyethylene terephthalate
Ph_3_P	Triphenylphosphine
