# Supplementary material for: A Review of Cinnamic Acid’s Skeleton Modification: Features for Antibacterial-Agent-Guided Derivatives
Source: Molecules. 2024 Aug 20;29(16):3929. doi: 10.3390/molecules29163929 (PMC11357405; doi:10.3390/molecules29163929)
Supplement: Supplementary file 1 [file molecules-29-03929-s001.zip › molecules-3139695-supplementary.pdf]

SUPPORTING INFORMATION

Review of Cinnamic Acid's Skeleton Modification: Features for Antibacterial-Agent-Guided Derivatives

Rose Malina Annuur [1], Desita Triana [2], Teni Ernawati [1], Yuta Murai [2], Muhammad Aswad [3], Makoto Hashimoto [2,*] and Zetryana Puteri Tachrim [1,*]

[1] Research Center for Pharmaceutical Ingredient and Traditional Medicine, National Research and Innovation Agency, Kawasan Sains Teknologi (KST) BJ Habibie, Serpong, South Tangerang 15314, Indonesia

[2] Division of Applied Bioscience, Graduate School of Agriculture, Hokkaido University, Kita 9, Nishi 9, Kita-ku, Sapporo 0608589, Japan

[3] Faculty of Pharmacy, Hasanuddin University, Makassar 90245, Indonesia; aswadfar@unhas.ac.id

* Correspondence: hasimoto@agr.hokudai.ac.jp (M.H.); zetr001@brin.go.id (Z.P.T.)

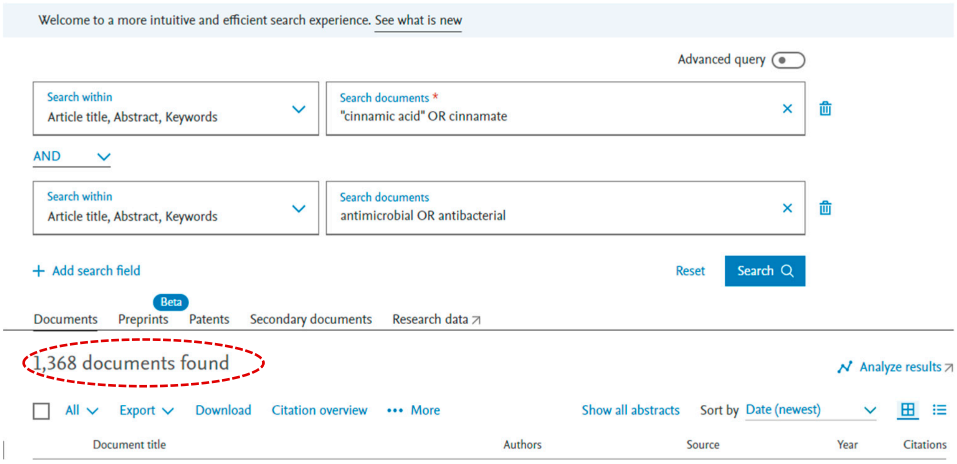
Welcome to a more intuitive and efficient search experience. See what is new
Advanced query
Search within
Article title, Abstract, Keywords
Search documents *
"cinnamic acid" OR cinnamate
AND
Search within
Article title, Abstract, Keywords
Search documents
antimicrobial OR antibacterial
Add search field
Reset
Search
Beta
Documents
Preprints
Patents
Secondary documents
Research data
1,368 documents found
Analyze results
All
Export
Download
Citation overview
More
Show all abstracts
Sort by
Date (newest)
Document title
Authors
Source
Year
Citations

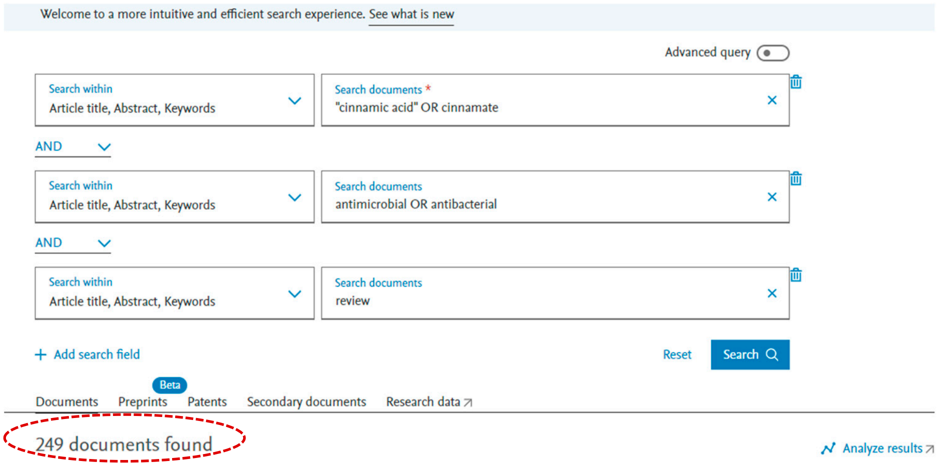
Welcome to a more intuitive and efficient search experience. See what is new
Advanced query
Search within
Article title, Abstract, Keywords
Search documents *
"cinnamic acid" OR cinnamate
AND
Search within
Article title, Abstract, Keywords
Search documents
antimicrobial OR antibacterial
AND
Search within
Article title, Abstract, Keywords
Search documents
review
Add search field
Reset
Search
Beta
Documents
Preprints
Patents
Secondary documents
Research data
249 documents found
Analyze results

Indexer/ Database	Query	Result Documents	Date of Search
Scopus	(TITLE-ABS-KEY ("cinnamic acid" OR cinnamate) AND TITLE-ABS-KEY (antimicrobial OR antibacterial))	1368	17th January 2024
	(TITLE-ABS-KEY ("cinnamic acid" OR cinnamate) AND TITLE-ABS-KEY (antimicrobial OR antibacterial) AND T ITLE-ABS-KEY (review))	300	
Limitation	Document type : review;<br>Source Type : Journal, Boo, Conf Preceeding<br>Language : English		
	(TITLE-ABS-KEY ("cinnamic acid" OR cinnamate) AND TITLE-ABS-KEY (antimicrobial OR antibacterial) AND T ITLE-ABS-KEY (review)) AND PUBYEAR > 1990 AN D PUBYEAR < 2024 AND (LIMIT-TO (DOCTYPE , "re")) AND (LIMIT-TO (SRCTYPE , "j") OR LIMIT-TO (SRCTYPE , "p") OR LIMIT-TO (SRCTYPE , "b")) AND (LIMIT-TO (LANGUAGE , "english"))	249	

Year	Documents	Year	Documents
2023	49	2006	1
2022	35	2005	1
2021	36	2004	2
2020	22	2003	1
2019	20	2002	1
2018	14	2001	0
2017	13	2000	0
2016	7	1999	1
2015	8	1998	0
2014	8	1997	0
2013	4	1996	0
2012	12	1995	0
2011	6	1994	0
2010	1	1993	0
2009	1	1992	0
2008	4	1991	1
2007	1		

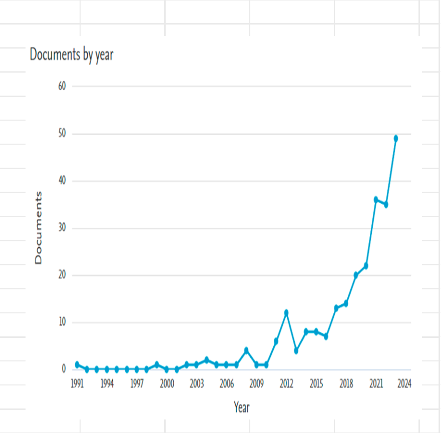

Figure S1. Scopus data*

*Keywords confirmation by the thesaurus INIS Repository Search - INIS Thesaurus (iaea.org) (https://inis.iaea.org/search/thesaurus.aspx) dan Library of Congress Subject Headings PDF Files: https://www.loc.gov/aba/publications/FreeLCSH/freelcsh.html.
Data collections on 17 January 2024.

Methods:
The present review manuscript considered the literature on specific keyword Figure S1 associated to full articles. Only literature available in these databases which match with cinnamic acid main skeleton was chosen especially focusing to its modification guided with antibacterial activity.
